# Effect of Conventional Adhesive Application or Co-Curing Technique on Dentin Bond Strength

**DOI:** 10.3390/ma14247664

**Published:** 2021-12-12

**Authors:** Josipa Vukelja, Eva Klarić Sever, Ivan Sever, Silvana Jukić Krmek, Zrinka Tarle

**Affiliations:** 1 Department of Endodontics and Restorative Dentistry, School of Dental Medicine, University of Zagreb, 10000 Zagreb, Croatia; eklaric@sfzg.hr (E.K.S.); jukic@sfzg.hr (S.J.K.); tarle@sfzg.hr (Z.T.); 2 Thinktourism, 10000 Zagreb, Croatia; ivan.sever@thinktourism.org

**Keywords:** dentin bond strength, shear bond strength, co-curing, reliability analysis

## Abstract

The aim of this in vitro study was to assess the effect of two different adhesive application methods on shear dentin bond strength (ISO 29022) using three various adhesive systems. A mid-coronal section of 77 intact third human molars with fully developed apices was made to create flat bonding substrates. The materials used in the study were Excite F (Ivoclar Vivadent), Prime&Bond Universal (Dentsply Sirona) and G-Premio Bond (GC). The application of each adhesion system was performed in two different ways. In the first group, the bonding agent was light cured immediately after the application (conventional method), while in the second group the adhesive and composite were cured concurrently (“co-curing” method). A total of 180 specimens were prepared (3 adhesives × 2 method of application × 30 specimens per experimental group), stored at 37 °C in distilled water and fractured in shear mode after 1 week. Statistical analysis was performed using ANOVA and Weibull statistics. The highest bond strength was obtained for Prime&Bond conventional (21.7 MPa), whilst the lowest bond strength was observed when co-curing was used (particularly, Excite F 12.2 MPa). The results showed a significant difference between conventional and co-curing methods in all materials. According to reliability analysis, the co-curing method diminished bond reliability. Different application techniques exhibit different bond strengths to dentin.

## 1. Introduction

The foundation of contemporary restorative dentistry is adhesion. A dental adhesive system as an intermediate material bonds restorative material to hard dental tissues and enhance resistance of tooth-restoration interface, retention and marginal sealing [1]. However, the greatest limitation in the field of resin composite and one of the main reasons for the clinical failure of today’s adhesion systems is polymerization stress [2]. During the curing process of composites resins, polymerization shrinkage occurs due to the conversion of monomers into polymers which results in a decreased overall volume. This can cause the development of internal contraction stresses and stresses at the margins of the restoration [3]. If contraction forces overcome the bond strength that leads to the marginal failure, respectively postoperative sensitivity, marginal microleakage, marginal staining, secondary caries and pulp damage [4]. Various factors influence the stress formation which includes configuration factor and cavity geometry, application technique and modulus of elasticity and stiffness of restorative material. Numerous clinical procedures and techniques have been suggested to reduce polymerization shrinkage and elevate the marginal sealing of restorations. One of the proposed methods is simultaneously light curing of the resin adhesive and first layer of the composite resin, i.e., co-curing technique [5,6,7,8,9,10,11,12]. Unterbrink and Liebenberg considered using flowable composites as filled adhesives, combining a single component adhesive as a dentin primer with a thin layer of flowable composite as a filled adhesive, both of which were cured together to eliminate oxygen inhibition of extremely thin adhesive layers [13].

Although there is a lack of studies on co-curing effect of on shear bond strength of resin composite to dentin, previous research has not provided evidence in favor of the co-curing method. Viswanathan et al., same as Chapman et al. reported significantly lower shear bond strengths to dentin when co-cured. They based the flaw on two factors: inadequate bonding agent curing and stress contraction of the curing overlapping composite resin [14,15].

The literature data suggest a possible success of co-curing technique if a bulk-fill resin composite is used. Bulk-fill resin composites have competence to transmit curing light much deeper than conventional composite materials which result in lower contraction stress [16,17,18,19]. Deliperi et al. reported that there was no difference between various adhesive placement techniques in reducing microleakage [20].

Nowadays, the technology of dental adhesive is progressing rapidly. Two step etch-and-rinse method, respectively fifth-generation of adhesives has becomes increasingly widespread in everyday clinical practice. A main concern connected with etch-and-rinse adhesives in terms of bond stability is that phosphoric acid may over-etch dentin, causing resin to penetrate deeper into the exposed collagen-fibril network than resin will be able to infiltrate in the application time. Consequently, it can lead to nanoleakage and bond degradation [21,22,23].

Universal adhesives as the newest eighth-generation of adhesives, may be used in either full etch-and-rinse, selective enamel etch or self-etch bonding modes, according to the dentist’s preferences or particular clinical situation [24]. Given the possibility of a shortened process, universal adhesives reduce the treatment time.

Furthermore, providing application diversity with the ability to adhere to indirect tooth restoration like a glass-rich and glass-poor zirconia ceramics [25,26]. When the shortcomings of universal adhesives are cited, it is often mentioned their small film thickness, often below 10 μm, permits oxygen to prevent polymerization of the adhesive layer for a large portion of its depth. The adhesive interface is insufficiently stabilized by suboptimal polymerization, reducing the adhesive layer’s capacity to absorb stress. Containing incorporated silane, by which universal adhesives can chemically bond to glass-rich ceramics potentially compromised bonding performance [27].

The purpose of this laboratory study was to evaluate dentin bond strength of a flowable “bulk fill“ composite resin associated with three various adhesives using different curing techniques. The following hypotheses were tested: (1) shear bond strength to dentin is not affected by using different bonding agents, (2) shear bond strength is not affected by adhesive application technique.

## 2. Materials and Methods

### 2.1. Dentin Substrate Preparation

**Figure 1:**
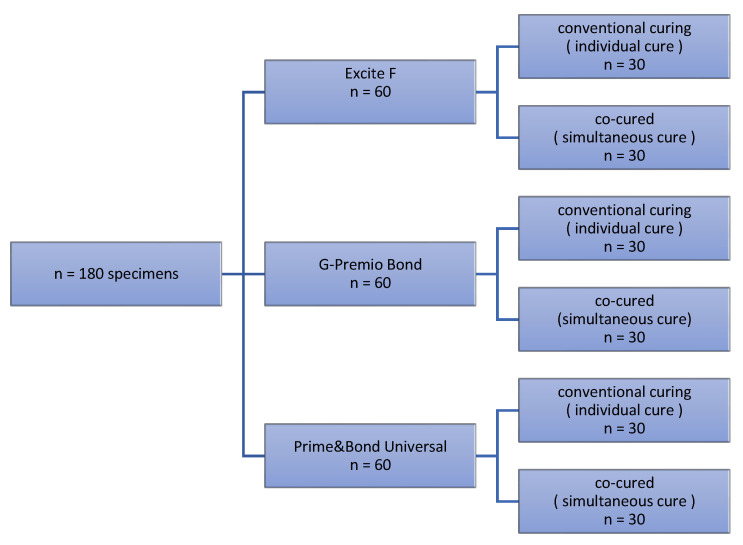
Flowchart of the groups used in the study.

Following extraction 77 noncarious human third molars with completed root formation were collected and stored at 1% chloramine solution. The teeth were utilized for maximum of 3 months after they were extracted. Dentin substrates were prepared by means of low-speed saw (IsoMet, Buehler; Lake Bluff, IL, USA) at 300 rpm with continuous watercooling. Sections were done in mid-coronal part to obtain two dentin slabs, tagged as the “occlusal“ and “radicular“ part. If their surface area was adequate to place more than one composite specimen, slabs were further cut. An average of 2.3 dentin slabs were obtained per tooth. Dentin samples were mounted in an Ultradent mold (Ultradent Products, South Jordan, UT, USA) using a cold-curing methacrylate resin (Technovit 4004, Kulzer, Germany). To create a flat bonding area, dentin surface was polished with 600-grit silicone carbide (SiC) paper (PRESI, Eybenes, France), rinsed thoroughly with water and instantly used for the bonding procedure. A total of 180 specimens were prepared and randomly assigned to 6 experimental groups (3 adhesives × 2 method of application) (Figure 1). The number of specimens per experimental group was *n* = 30, taking care that only one section of one tooth is in the same experimental group.

### 2.2. Bonding Procedure

Materials used in this study were three different adhesives and single bulk-fill composite (Table 1). After the slabs were divided into experimental groups, surface of the dentin was slowly dried with air as far as no visible moisture was left. A polymer adhesive strip with a hole of 2.4 mm in diameter and thickness of 0.2 mm was used to define the bonding area. Each adhesive was applied in two different ways. In the first method, the adhesive was light cured according to the manufacturer’s instructions (Table 2) before adding the resin composite (conventional method). Composite cylinders (2.38 mm internal diameter and 2.0 mm height) were formed on the adherent surface using a bonding clamp and plastic mold inserts (Ultradent Products, South Jordan, UT, USA). The resin composite was placed into the mold, and light cured for 20 s. In the second method, the adhesive was light cured simultaneously with the added resin composite (co-curing). Adhesive systems were used according to manufacturers’ instructions but they did not light cured immediately after application. After the adhesive system was applied in a single layer, it was air dried slowly but not immediately light cured. The bonding agent was light cured with composite resin for 40 s as in some of the previous studies [1,4,14,28]. Light curing, for both methods, was performed with one curing unit from a distance of 1 mm (Bluphase Style, Ivoclar Vivadent; Schaan, Liechtenstein, Ser. No. 1120006563) with a high light intensity of 1100 mW/cm^2^ which was measured using LED curing light radiometer Bluephase Meter II (Ivoclar Vicadent, Schaan, Liechtenstein). After removal of the mold, specimens were stored in distilled water in an incubator (INEL, Zagreb, Croatia) at 37 °C for one week.

### 2.3. Shear Bond Strength Testing

After one week of storage, specimens were fractured in a macroshear mode by loading the specimens in bond strength testing machine UltraTester (Ultradent Products, SAS Institute Inc., Cary, NC, USA). Testing was performed at a constant crosshead speed of 1 mm/min until fracture, i.e., until the composite cylinders became de-bonded from the dentin surface. The shear bond strength values of the adhesives to dentin were regulated in accordance with ISO 29022 [29].

### 2.4. Weibull Analysis

Reliability analysis begins by ranking the samples according to the calculated bond strength. The values which are plotted along the horizontal axis of a Weibull graph are obtained as natural logarithms of bond strength. The following equation gives the probability of failure (*Pf*) for each specimen from a set of n specimens:

(1) $$\;\;\;\;\;Pf=\frac{i-0.5}{n}$$

where *i* is the ranking number in ascending order of bond strength data (weakest rank 1, strongest rank N) while *n* is the total number of specimens inside the experimental group. On the vertical axis is plotted double natural logarithm of [1/(1−Pf)]. Maximum likelihood estimation is then used to fit a function through the plotted data points. The obtained slope represents the Weibull modulus. The Weibull modulus, or shape parameter, reflects variability of bond strength measure. Lower value of *m* points to a wider distribution of defects and less predictable bond strength. Higher value of *m* (i.e., steeper slope of fit line) denotes higher reliability. The second parameter of Weibull distribution is characteristic bond strength, i.e., the scale parameter which is the value taken from the *x*-axis, where the probability of failure (*Pf*) on the *y*-axis is equal to 63.2%. The decline in the scale parameter shifts the data to the left, toward the lower values of ln(strength) on the *x*-axis [30].

## 3. Results

**Figure 2:**
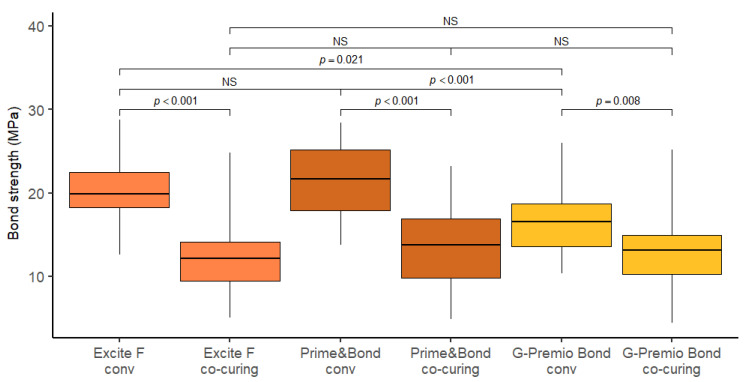
Box plots of shear bond strength. Note: Horizontal line in each box displays the mean value, boxes display the interquartile range (distance between the upper and lower quartiles), and whiskers display the range of data (distance between the highest and lowest data points).

The distribution of bond strength values is graphically represented by Box-plot diagrams (Figure 2). On average, the highest bond strength values were recorded using Prime&Bond conventional application method (21.7 MPa), and the lowest using Excite F after co-curing (12.2 MPa).

The distribution of bond strength values was generally somewhat higher with the conventional application method compared to co-curing. The measurements of bond strength for co-curing were more variable (coefficient of variation between 34% and 40%) compared to conventional bonding (coefficient of variation between 20% and 24%). On average, the largest difference in bond strength was recorded between the conventional and co-curing techniques when using Prime&Bond and Excite F materials. For Prime&Bond, the bond strength was on average higher by 8.14 MPa (*p* < 0.001) with conventional application compared to co-curing, and by 7.63 MPa (*p* < 0.001) for Excite F. Higher bond strength of conventional application was also observed when using G-Premio BOND material, but the effect was somewhat smaller (3.65 MPa; *p* = 0.008). The comparison of materials showed a statistically significantly higher strength in conventional application on Prime&Bond compared to G-Premio Bond (on average higher by 4.91 MPa; *p* < 0.001), and on Excite F material compared to G-Premio Bond (3.27 MPa; *p* = 0.021).

**Figure 3:**
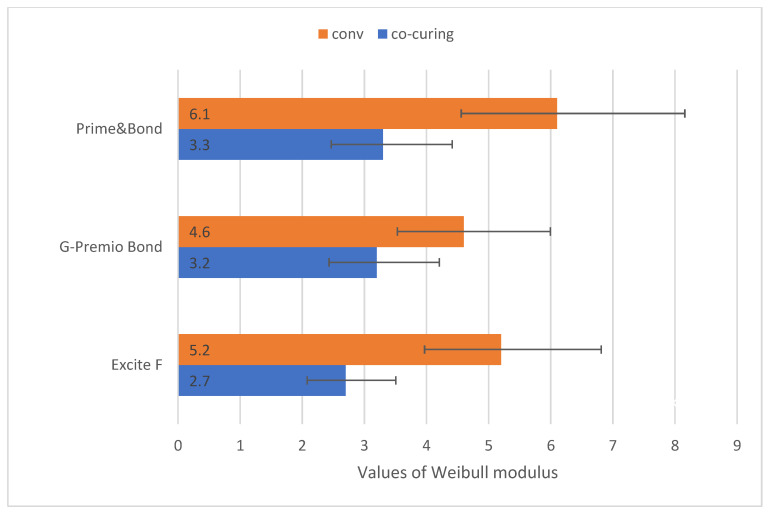
Weibull modulus. Note: Error bars represent 95% confidence intervals.

According to Figure 3, the Weibull modulus was higher for conventional application, for all three materials included in the study. Thereby, compared to conventional applications, the reliability of co-curing is lower.

**Figure 4:**
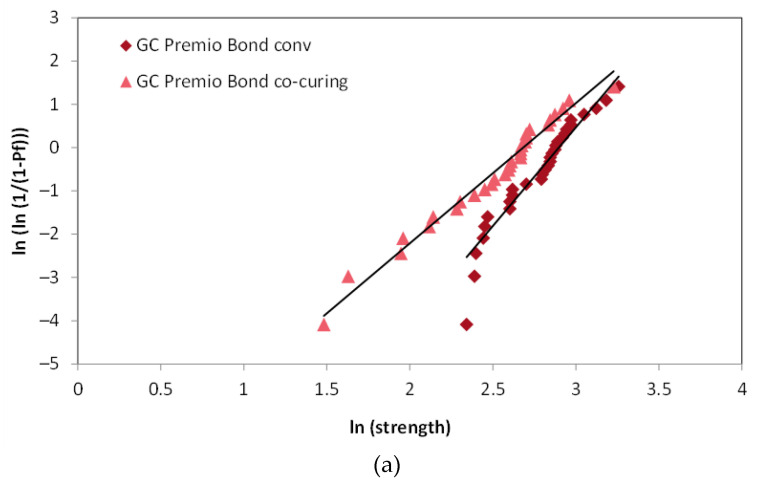

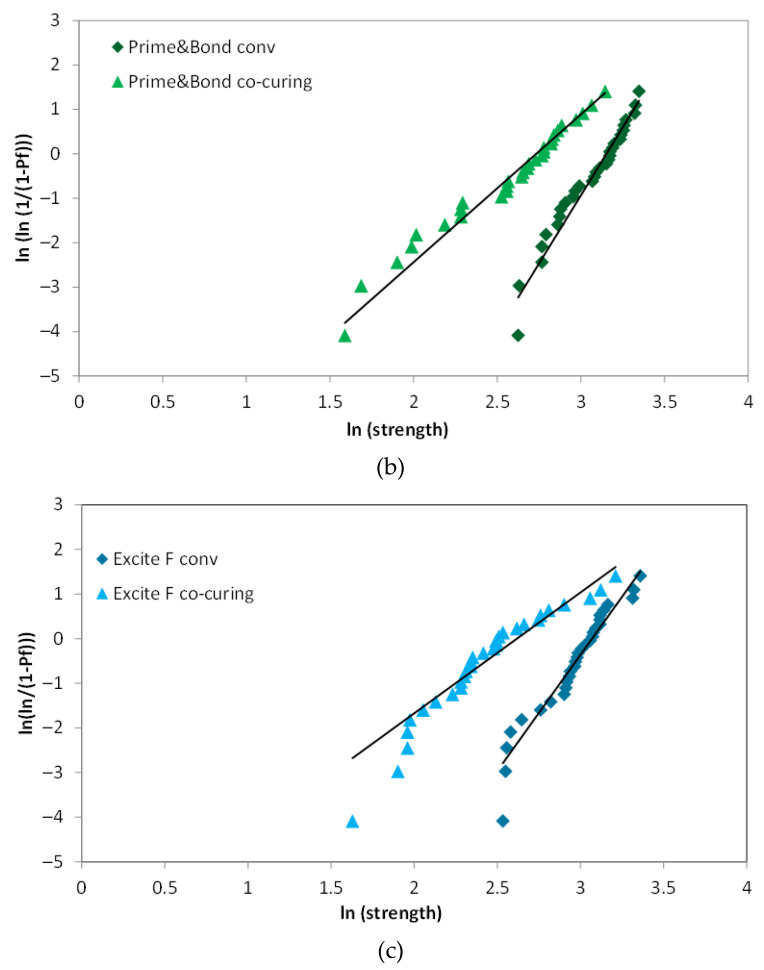
Weibull diagrams for the conventional and co-curing method of application for all three adhesive systems used in the study. Slopes of the fit lines represent Weibull modulus. (**a**) GC Premio Bond, (**b**) Prime&Bond, (**c**) Excite F.

**Figure 5:**
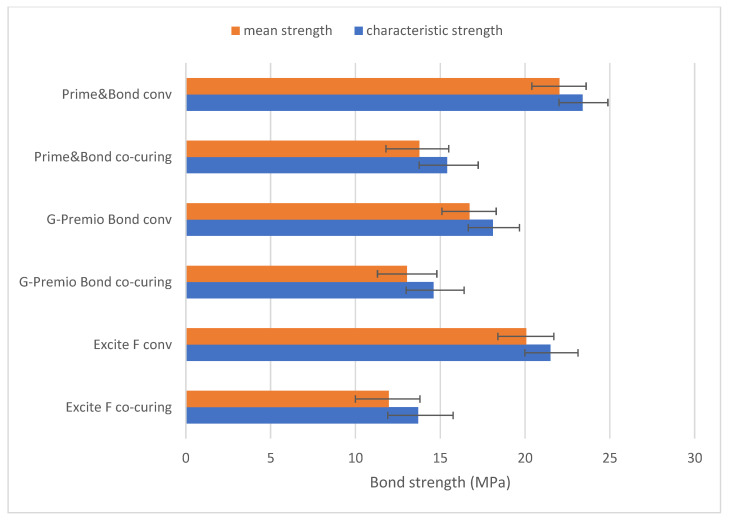
Comparison of characteristic strength (*Pf* ≈ 63.2%) and mean strength (*Pf* = 50%) across experimental groups. Note: Error bars represent 95% confidence intervals.

Weibull diagrams in Figure 4 show that conventional application of adhesive shifted the Weibull distributions towards higher values on the *x*-axis, reflecting the increase in the scale parameter, while the steeper slope of the fit lines reflects a higher consistency and less scattering of strength values. The mean strength (*Pf* = 50%) and characteristic strength (*Pf* ≈ 63.2%) followed a similar variation pattern across all experimental groups (Figure 5).

The bond strength between the occlusal parts of the tooth (mean = 17.1 MPa) and the radicular parts of the tooth (mean = 15.3 MPa) was statistically significantly different (*p* = 0.007).

## 4. Discussion

Adhesive systems are often selected based on the results achieved by laboratory testing, but it should be kept in mind that these tests are influenced by various factors such as test specimen properties, preparation of specimens, handling of materials, specimen storage, experimental setup design, and experimental technique. Although the shear test method is the most often used bond strength technique [31], some researchers deem that it has little value in predicting the clinical performance of dental adhesives, and the distribution of stress is not as homogeneous as in a microtensile mode [32,33]. In addition to the shear method, objections can also be found to the tensile test; such as that restorations are practically never loaded in tensile mode [34].

Therefore, all mentioned loading tests have their values and limitations. In order to standardize the shear test protocol [35], Ultradent jig (Ultradent, Salt Lake City, UT, USA) has been made. This specific ultradent jig comes in contact with a larger sample surface; wrapping around the composite material and surrounding half of the specimen. Thus device allocates stress over a larger area, resisting higher load levels. In this way, shear bond strength can be more precisely evaluated [36,37]. Information drawn from the literature often mention that a parameter ˝limitation of the bonding area is important“, listed in the ISO Technical Specification (TS) with the title “Testing the adhesion to tooth structure” (No. 11405, first edition 1994, second edition 2003, third edition 2015), is most frequently skipped [33,37,38,39]. In our study, the bonding area was defined and limited.

One of the variables affecting bond strength test results is dentin depth, as it is influenced by water content and the diameter of dentinal tubulus [40,41,42]. In this study, the difference in dentin depth was 0.2 mm, which was the thickness of the diamond cutting saw used to separate the tooth into occlusal and radicular slabs midcoronally. A statistically significant difference in bond strength between the occlusal and the radicular parts of the tooth was observed in this study. Although there are underlying assumptions in favor of this result, it is important to emphasize that the number of samples per group was too small for an accurate estimate.

As previously stated, laboratory evaluation of the co-curing technique applied to tooth dentin assessed negative results. Chapman et al. compared shear bond strength of three self-etch adhesives to enamel and dentin on permanent teeth, while Viswanathan et al. evaluated shear bond strength to primary enamel and dentin using one total-etch and one self-etch adhesive. Both studies observed no significant effect among different methods of application on enamel bond strength, but greater dentin strength values when application was conventional [14,15].

Earlier research of McCabe and Rusby also did not go in favour of the co-curing method when used on dentin [43]. The conclusion regarding bond strength was not concise and did not point a clear explanation of their findings, but they relied on the various chemical composition, microstructure and chemical reactivity of enamel to adhesives in comparison to those of dentin, since on enamel co-curing did not show worse results.

These findings referred to the additional effect of the composite resin shrinkage. During simultaneous light polymerization of adhesive and composite, penetration of resin tags into dentinal tubules and their lateral branches are deteriorate because composite resin tends to shrink [44]. Since there is no complete creation of a network of interconnected resin tags, an insufficiently strong bond is created [45]. Furthermore, due to probable attenuation and dispersion of light energy via the overlaying composite, the restricted exposure of resin adhesive to the curing light may result in inadequate and unfinished curing of the resin adhesive, which will most likely remain in the gel form [46].

The length of the polymer chains is determined by the amount to which the adhesive monomers are transformed into a polymer, which in turn defines the polymer’s final strength [47].

Hence, due to unsatisfying polymerization a decrease in the physical/mechanical properties of resin composites can occur [48]. In comparison to previously mentioned investigations, measurements made in our study gave us the opportunity to compare different materials (different adhesive systems and conventional composites), special testing machine and location of specific tooth surface which makes this study original.

Bulk-fill composites can be adequately polymerized at a thickness of 4 mm [16,17,18]. Some studies showed a possible depth of cure up to 5.5 mm [49]. The composition of bulk-fill composite resins is different from commercial ones in regard to fillers, photoinitiators and monomers [50,51]. Further, bulk fill materials result in having less shrinkage and lower values of contraction stress in comparison to the conventional types of composite resins [52]. The recorded lower bond strength values of the co-cured adhesive to dentin in comparison to the conventional one came in total agreement with the results of Abdelaziz and Saleh study. They evaluated the influence of adhesive application modalities on their bonding values using several different composites, among which was a bulk-fill composite [19]. Despite the overall reduced bond strengths of co-cure application technique, the bonding values of the bulk-inserted resin composites appeared to be comparable or even higher than those of the incrementally-inserted resin composite. However, the difference between mentioned study and the current study could be referred to the different types of human teeth (premolars), different testing machines (universal testing machine: model 5965, Instron, Grove City, PA, USA) and in addition to the different types of materials (adhesive and composite resin) utilized in their study.

Bulk-fill composite materials transmit light better than typical composites, according to Bucuta and Ilie [16]. Due to light attenuation, the deeper layers still not attain optimal polymerization as the surface, despite their higher translucency [53,54].

The following hypotheses were tested: (i) the application adhesive procedure has no effect on shear bond strength and (ii) using various bonding agents has no effect on the shear bond strength to dentin.

The null hypothesis of the current study denied any influence of co-curing technique compared to conventional adhesive application on dentin bond strength, as well as different bonding agents has no effect on shear bond strength to dentin. The study’s hypothesis was rejected. As stated above, a significant effect of the method of adhesive’s application was clearly noticed on the bond strength. Results of the current study showed lower bond strength of co-cured adhesives to dentin. Further, the comparison of materials showed a statistically significantly higher strength in conventional application on Prime&Bond and Excite F compared to G-Premio Bond. The reason for that could be low pH of G-Premio Bond. There are claims that aside from micromechanical retention, some mild adhesives with higher pH can chemically interact with the hydroxyapatite calcium that covers the collagen in partially demineralized dentin [55,56,57]. In some instances, an error may have occurred during sample preparation. G-Premio Bond use solvent is acetone which has the ability to draw water out of the substrate. When it is applied over a dry dentin, however, the collapse of collagen fibrils cannot be avoided, unlike when water is used as a solvent [58,59]. The wet-bonding procedure is used to apply acetone-containing adhesives to remove water [60]. It is likely that a hybridoid layer developed during the preparation of dentinal specimens due to an overdrying phenomena [59,61].

The bond strength test findings are influenced by a variety of factors, resulting in a wide range of outcomes [62]. The brittle nature of materials like dental adhesives and composites, on the other hand, is the primary cause of variability [63]. Even when a group of apparently similar specimens is tested under the same conditions, the maximum stress brittle materials can withstand varies unexpectedly from specimen to specimen [64]. Brittle materials’ strength is already defined by the flaws or pre-existing imperfections present in the sample, therefore its measured strength is based on the likelihood of a critical defect occurring in their structure [30,65]. The most common way of representing bond strength testing data, which are made up of a series of measurements taken on a number of samples that appear to be identical [63,66], is to provide the number of tests completed, the mean strength, and the standard deviation [36,67,68]. This model implies that the mean value is the “real value”, and that data scattering about this actual value is attributable to test methodology or specimen preparation differences. When the fracture process is brittle, the findings reveal a lot of variance, which is due to the features of the examined specimens rather than the material itself [63,66]. As a result, several authors recommend that brittle materials should be classified based on the likelihood of failure at a given stress level, which may be estimated using the Weibull distribution function [39,65,66,69]. Weibull statistics produced the same findings as conventional statistics in our investigation, with the exception that characteristic strength values were somewhat higher than mean strength.

## 5. Conclusions

Under the limitations of this in vitro study, it is possible to conclude that the adhesive application method influences the shear bond strength of dentin. In all of the adhesion systems tested, the co-curing technique resulted in lower bonding strength values in regards to a conventional method. As a result, shortening bonding methods (by ignoring the manufacturer’s specifications) weakens the bond. Additional experiments are needed, such as comparisons of conventional and bulk composites in different application approaches and analyzing the bond strength of co-curing specimens over different time periods.

## Figures and Tables

**Table 1:** Materials used in this study.

Material	Type	Chemical Formulation *	pH	Manufacturerand LOT No.
Total Etch	Etchant	phosphoric acid (37 wt.% in water), thickening agent and colour pigments		Ivoclar Vivadent AG, Schaan, Liechtenstein LOT: Y39066 EXP: 2022-01
Excite F	Adhesive (total-etch)	HEMA, Bis-GMA, UDMA, Phosphonic acid acrylate, highly dispersed siliconedioxide, initiators, stabilizers and potassium fluoride in an **ethanol solution**, camphorquinone, trimethylbenzoyldiphenylphosphine oxide	2.5	Ivoclar Vivadent AG, Schaan, Liechtenstein LOT: Z020C1 EXP: 2023-11
G-Premio Bond	Adhesive (universal)	10-MDP, 4-MET, MDTP, methacrylic acid ester, silica, **acetone, water**, photoinitiators	1.5	GC Corp., Tokyo, Japan LOT: 1906132 EXP: 2021-06
Prime&Bond Universal	Adhesive (universal)	Bi- and multifunctional acrylate, 10-MDP, PENTA, phosphoric acid modified acrylate resin stabilizer, **isopropanol**, camphorquinone /tertiary amine	>2.5	Dentsply Sirona, Konstanz, Germany LOT: 2009000399 EXP: 2022-08
SDR Plus Bulk Fill Flowable	Bulk fill flowable resin composite	Polymerizable dimethacrylate resins, polymerizable UDMA, barium boron fluoro–alumino-silicate glass, silicon dioxide, titanium dioxide, synthetic inorganic iron oxides, photoinitiators		Dentsply Sirona, Konstanz, Germany LOT: 00028647 EXP: 2022-08

* According to the manufacturers’ information. HEMA: 2-hydroxyethyl methacrylate, BisGMA: Bisphenol-A glycidyl methacrylate, UDMA: urethane dimethacrylate, 10-MDP: 10-methacryloyloxydecyl dihydrogenphosphate, 4-MET: 4-methacryloxyethyl trimellitic acid, MDTP: methacryloyloxydecyl dihydrogen thiophosphate, PENTA: dipentaerythritol pentaacrylate monophosphate.

**Table 2:** Manufacturers’ instructions.

Excite F	1. Apply the etchant on the dentin for 15 s.
	2. Remove all etchant gel with a water rinsing for at least 5 s.
	3. Excess moisture should be removed leaving the dentin surface with a glossy wet appearance (wet bonding)—Do not overdry the dentin!
	4. Disperse to a thin layer with a weak stream of air, thereby removing any excess.
	5. Polymerize for 10 s at a light intensity of more than 500 mW/cm^2^.
G-Premio Bond	1. Apply the adhesive and leave for 10 s.
	2. Dry thoroughly with air under maximum air pressure for 5 s.
	3. Light cure for 10 s.
Prime&Bond Universal	1. Apply slightly on dentin for 20 s.
	2. Dry thoroughly with air under maximum air pressure for 5 s.
	3. Light cure for 10 s (≥800 mW/cm^2^).

## Data Availability

The datasets generated and analyzed during the current study are available from the corresponding author on reasonable request.
